# CircGNB1 facilitates the malignant phenotype of GSCs by regulating miR-515-5p/miR-582-3p-XPR1 axis

**DOI:** 10.1186/s12935-023-02970-2

**Published:** 2023-07-05

**Authors:** Jinpeng Hu, Guoqing Zhang, Yongfeng Wang, Kai Xu, Lian Chen, Gang Luo, Jinkun Xu, Hao Li, Dongmei Pei, Xiang Zhao, Zhengting Guo, Xinqiao Li, Shengliang Zong, Yang Jiang, Zhitao Jing

**Affiliations:** 1 Department of Neurosurgery, The First Hospital of China Medical University, No. 155 North Nanjing Street, Shenyang, 110001 People’s Republic of China; 2 Department of Neurosurgery, Shanghai Tenth People’s Hospital, Tongji University School of Medicine, Shanghai, 200072 People’s Republic of China; 3 Department of Radiology, The First Hospital of China Medical University, Shenyang, People’s Republic of China; 4 Liaoning Maternal and Child Health Hospital, No. 240 Shayang Road, Shenyang, 110005 People’s Republic of China; 5 Department of Health Management, Shengjing Hospital of China Medical University, Shenyang, 110004 People’s Republic of China

**Keywords:** Glioma, circGNB1, Glioma stem cells, XPR1, Malignant progression

## Abstract

Glioma is the most common and aggressive primary malignant brain tumor. Circular RNAs (circRNAs) and RNA-binding proteins (RBPs) have been verified to mediate diverse biological behaviors in various human cancers. Therefore, the aim of this study was to explore a novel circRNA termed circGNB1 and elucidate relative molecular mechanism in functional phenotypes, which might be a potential prognostic biomarker and therapeutic approach for glioma. CircGNB1 was upregulated in glioma and closely associated with the low poor prognosis. Functional assays demonstrated that circGNB1 overexpression promoted glioma stem cells (GSCs) viability proliferation, invasion, and neurosphere formation. Mechanistically, circGNB1 upregulated the expression of oncogene XPR1 via sponging miR-515-5p and miR-582-3p. The following experiments proved XPR1 could promote the malignant phenotype of GSCs via upregulating IL6 expression and activating JAK2/STAT3 signaling. Moreover, the RNA binding protein IGF2BP3 could bind to and maintain the stability of circGNB1, thus promoting the effects of circGNB1 on GSCs. Our study reveals that circGNB1 plays a crucial role in promoting tumorigenesis and malignant progression in glioma, which provides a promising cancer biomarker.

**Supplementary Information:**

The online version contains supplementary material available at 10.1186/s12935-023-02970-2.

## Background

Glioma is the most common primary intracranial malignant tumor in the brain with poor prognosis [1]. However, conventional therapies, including surgery, radiotherapy and chemotherapy, are hardly able to improve the low survival of patients with glioblastoma multiforme (GBM) in particular [2, 3]. Moreover, glioma stem cells (GSCs) play a critical role in tumor initiation, malignant progression, multiple differentiation and recurrence, so they are closely related with glioma cell proliferation, invasion, and neurosphere formation [4, 5]. Hence, to further explore novel therapeutic target and elucidate the fundamental molecular mechanisms may provide new sights and potential strategies for GBM therapy.

Circular RNAs (circRNAs) are produced by RNA back splicing and covalently characterized as closed-loop structures containing a typical 3′–5′ phosphodiester bond [6, 7]. Due to the special circular structures, circRNAs are more stable and resistant to degradation by exonucleases than their linear counterparts. More and more evidence has claimed that circRNAs play vital roles in the tumorigenesis and biological development in various types of cancers, including glioma [8]. For example, the circACTN4 could promote tumorigenesis and progression of breast cancer [9]. Previous studies have showed that circRNAs can facilitate physiological and pathological processes via sponging microRNA (miRNA) and protein, interacting with RNA-binding proteins (RBPs), regulating transcription, or translating peptides and proteins [10, 11]. Previous study revealed that circGNB1 could facilitate triple-negative breast cancer progression by sponging miR-141-5p [12]. However, the functions and underlying mechanisms of circRNAs are not completely clear in gliomas.

MiRNAs are a class of endogenous non-coding RNAs with a length of 18–23 nucleotides approximately, and they can downregulate the target mRNAs by suppressing translation or facilitating degradation [13]. CircRNAs can further regulate miRNA-targeted gene expression by binding to miRNA response elements (MREs), which acts as competing endogenous RNA (ceRNA) [14, 15]. In addition, studies have shown that RBPs can participate in the regulation of post-transcription levels and interact with circRNAs, affecting their transcription, translation, and formation [16]. IGF2BP3 (Insulin-like growth factor 2 mRNA binding protein 3) protein has been reported as an oncogene protein associated with glioma [17]. Moreover, previous study reported that XPR1 could promote progression of tongue squamous cell carcinoma via NF-κB signaling, which indicated that XPR1 may also act as an oncogene related to cancer [18].

Recently, GNB1 was reported as a novel oncogene in several cancers, such as cervical squamous cell carcinoma, retinoblastoma, and lung cancer. At first, we aimed to study whether GNB1 promoted the malignant proliferation of glioma [19–21]. However, our results revealed that GNB1 did not facilitate glioblastoma progression significantly. Due to the increasing studies about novel circRNAs in kinds of malignant cancers, we furtherly studied the possible circRNAs derived from GNB1 and identified a novel circRNA termed circGNB1, which was overexpressed in glioblastoma and promoted malignant progression of GSCs. Our study found circGNB1 was a novel and important oncogene which deserves further investigation in glioma, which may function biologically and become a promising therapeutic candidate as a cancer biomarker.

## Materials and methods

### Patient samples and ethical authorization

All clinical specimens of glioma patients (n = 70) were collected in the First Affiliated Hospital of China Medical University from January 2008 to October 2012. According to the WHO classification of tumors in the central nervous system (2007), glioma specimens were divided into three groups: Grade II (n = 20), Grade III (n = 25), and Grade IV (n = 25). Another 10 normal brain tissues (NBT) resected from patients who were non-glioma diseases were used as a negative control group. More detailed information is presented in Table 1. All patients signed informed consent, which was authorized by the Ethics Committee of the First Affiliated Hospital of China Medical University.

**Table 1:** Relationship of circGNB1 expression to clinical features of glioma patients

Clinical features	Samples (*n* = 70)	CircGNB1 expression^a^		*P*-value
		Low (*n* = 35)	High (*n* = 35)	
Sex				
Male	34	16	18	*P* = 0.773
Female	36	19	17	
Age				
≤ 50	26	12	14	*P* = 0.251
> 50	44	23	21	
WHO grade				
II	20	14	6	*P* < 0.001
III	25	17	8	
IV	25	4	21	
IDH status				
Wild	34	8	26	*P* = 0.002
Mutant	36	27	9	
1p/19q status				
Codeletion	37	26	11	*P* = 0.013
Non-codeletion	33	9	24	
H3F3A status				
Wild	38	24	14	*P* = 0.045
Mutant	32	11	21	
MGMT status				
Methylation	41	29	12	*P* = 0.018
Unmethylation	29	6	23	

^a^CircGNB1 expression was detected by qRT-PCR and ranked from low to high. The high expression of circGNB1 was defined as the expression level higher than the median expression level of circGNB1

### Cell culture and GSCs isolation

Human glioma cell lines (U87 and U251) were obtained from American Type Culture Collection (ATCC, Manassas, VA, USA) and cultured in Dulbecco’s Modified Eagle’s Medium (DMEM; HyClone, Logan, UT, USA) supplemented with 10% fetal bovine serum (FBS, Gibco, Carlsbad, CA, USA) and 1% penicillin/streptomycin (Gibco) at 37 °C in a humidified chamber with 5% CO_2_.

Meanwhile, six patient-derived primary glioma stem cells were isolated from WHO grade IV patients. The neurospheres cultures were performed as previously described [22]. Specifically, freshly resected glioma tissues were dissociated into single cells and cultivated in serum-free DMEM with 2% B27, 20 ng/mL recombinant human (rh) basic fibroblast growth factor (rh-FGF) and 20 ng/mL rh-epidermal growth factor (rh-EGF) (Gibco, Gaithersburg, MD, USA).The glioma stem cell markers CD133 (#ab216323, Abcam Technology, Cambridge, UK) and nestin (#ab105389, Abcam) were detected by immunofluorescence, and the multi-lineage differentiation capacity of GSCs was detected by immunofluorescence staining of GFAP (#ab7260, Abcam) and βIII tubulin (#ab18207, Abcam). All glioma cell lines and GSCs above were cultivated with no more than 20 generations for the experiment. Due to the suspension neurospheres of GSCs, we firstly digested spherical GSCs to individual cells before performing relative functional assays including Edu and invasion assays.

### Lentiviral vector construction and transfection

The lentivirus-based vectors for overexpression of circGNB1, XPR1, and IGF2BP3, along with RNAi-mediated knockdown of circGNB1, XPR1 and IGF2BP3, were constructed by Gene-Chem (Shanghai China). Meanwhile, the miR-582-3p/miR-515-5p mimics, inhibitors and their corresponding negative controls were purchased from Thermo Fisher Scientific (Assay ID: MC15598, MH15598, MC10387, AM10387, 4464058, 4464076, Thermo Fisher Scientific, Waltham, MA, USA). All the sequences applied for siRNAs are listed in Additional file 4: Table S1. The lentivirus transfection and efficacy measurements were carried out as previously described [22].

### Real-time quantitative reverse transcription PCR (qRT-PCR)

Real-time PCR was performed as previously described [22]. The total RNA of glioma tissues and GSCs was picked up by the Mini-BEST Universal RNA Extraction kit (TaKaRa, Kyoto, Japan). For circRNA and mRNA, reversed transcription was conducted according to the SYBR Green Master Mix (TaKaRa) via PCR LightCycler480 (Roche Diagnostics, Basel, Switzerland). For miRNA, cDNA was formed by the PrimeScriptTM RT nreagent kit (TaKaRa, Shiga, Japan). The miR-582-3p/miR-515-5p expression levels were detected by the TaqMan Universal Master Mix II (Assay ID: 002399, 001112. Thermo Fisher Scientific). Their expressions were normalized to endogenous control β-actin and fold change was determined as 2^−△△Ct^ in gene expression. The primers used in this study are listed in Additional file 5: Table S2.

### RNase R treatment

Total RNA was incubated with or without RNase R (3U/mg, Epicentre Technologies, Madison, USA) for 30 min at 37 °C. RNase R was used to confirm the existence of circGNB1 and eliminate the impact of linear GNB1.

### Western blotting

Western blotting was carried out as previously described [22]. Briefly, total protein from tissues and cells was extracted by using a total cell protein extraction kit (KeyGen Biotechnology, Nanjing, China). Then, equivalent amounts of protein were separated by SDS-PAGE and transferred onto the polyvinylidene difluoride (PVDF) and blocked with 2% bovine serum albumin (Beyotime Biotechnology, Beijing, China) for 2 h. The membrane were incubated overnight at 4 °C with the primary antibodies: β-actin (1:2000; #20536-1-AP, Proteintech), XPR1 (1:1000; #14174-1-AP, Proteintech), IL6 (1:1000; #ab233551, Abcam), IL6 (1:1000; #ab233551, Abcan), p-JAK2 (1:500; #WL02997, Wanleibio, Shenyang, China), JAK2 (1:500; #ab195055, Abcam), p-STAT3 (1:1000; #WLP2412, Wanleibio), STAT3 (1:2000; #ab76315, Abcam), IGF2BP3 (1:1000; #14642-1-AP, Proteintech), followed washing with PBS/T (Phosphate Buffer Solution with 0.05% Tween20). Next, the membranes were incubated with horseradish peroxidase-linked secondary antibody (1:1000; #SA00001-2, Proteintech) at room temperature for 1 h. Finally, immunoreactive bands were displayed by a chemiluminescence ECL kit (Beyotime Biotechnology, Beijing, China) and quantified by Image J software (National Institutes of Health, Bethesda, MD, USA) while the β-actin was used as internal control.

### Immunohistochemistry (IHC)

IHC was carried out as previously described [22]. In brief, the mouse brain tumor tissues were fixed with 4% paraformaldehyde first and embedded in paraffin. Then, paraffin-embedded tissues were cut into 4 μm sections which were incubated with the specific primary antibody against XPR1, IGF2BP3, IL6, Ki67 (1:100; #ab92742, Abcam), CD133 (1:100; #ab216323, Abcam) and nestin (1:100; #ab105389, Abcam). The images were captured using an optical microscope (Olympus, Tokyo, Japan), and the German immunohistochemical was applied to staining intensity.

### Immunofluorescence (IF)

Immunofluorescence staining was performed as previously described [22]. Briefly, the GSCs were incubated with primary antibody against CD133, GFAP, nestin or βIII-tubulin (1:100; Abcam) at 4 °C overnight. Then, the rhodamine-conjugated secondary antibody was used for multicolor immunofluorescence imaging, while DAPI solution was used for nuclear counterstaining. Finally, a laser scanning confocal microscope (Olympus) was used to visualize the staining.

### Cell viability assay

The Cell viability assay was performed as described previously [22]. Briefly, a Cell Titer 96^®^ Aqueous Non-Radioactive Cell Proliferation Assay Kit (Promega, Madison, WI, USA) was used to detect the cell viability according to the manufacturer’s instructions. Firstly, seed the GSCs into 96-well plates at a density of 1 × 10^3^ cells/well in triplicate. Then, the GSCs were incubated for 0, 24, 48, 72, 96, and 120 h.

### 5-Ethynyl-20-deoxyuridine (EDU) assay

The EDU assay was performed as described previously [22]. In short, cells (1 × 10^5^) were seeded in 24-well plates and cultured for 20 h. After incubation with EDU reagent for 2 h, cells were fixed with 4% paraformaldehyde (solarbio) and permeabilized with 0.5% Triton X-100 (solarbio). Sequentially, cells were counterstained following the manufacturer’s instructions of the EDU assay kit (Beyotime, Biotechnology, China). The images were captured using a laser scanning confocal microscope (Olympus) and then the EDU-positive cells percentage could be calculated.

### Luciferase activity analysis

Luciferase reporter assays were performed as previously described [22]. In brief, the luciferase reporter plasmids (circGNB1-wt and circGNB1-mt, XPR1-3′-UTR-wt and XPR1-3′-UTR-mt) were constructed by Gene-Chem (Shanghai, China). The GSCs were plated in wells of 96-well plates at a density of 5 × 10^3^ cells/well and co-transfected with the plasmids above for 48 h. Next, the Dual-Luciferase Reporter Assay System (Promega, USA) was used to detect the luciferase activity of tumor cells following the manufacture’s protocol.

### Neurosphere formation assay

The neurosphere formation assay was carried out as previously described [22]. Briefly, seed the GSCs in 24-well plates under the condition of 200 cells/well in a fresh medium for 7 days. Next, observe the relative neurosphere size via the optical microscope (Olympus).

### In vitro limiting dilution assay

The GSCs were seeded in 96-well plates under different condition of 1, 10, 20, 30, 40, or 50 cells/well, meanwhile each density was replicated for 10 times. After incubation for 7 days, count the neurospheres number and calculate the neurosphere formation efficiency via the Extreme Limiting Dilution Analysis (http://bioinf.wehi.edu.au/software/elda) [22].

### RNA immunoprecipitation (RIP) assay

The RIP assay was performed using RNA Immunoprecipitation Kit (Sigma, USA) in accordance with the manufacturer’s instructions. The GSCs were lysed in RIP buffer and then incubated with magnetic beads conjugated with anti-XPR1 antibodies, negative control IgG, or anti-AGO-2. The immunoprecipitated RNAs could be recovered after incubation with Proteinase K buffer (Omega, Shanghai, China). At last, qRT-PCR was used to check the relative expression in the precipitants.

### RNA pull-down assay

The coupling ability of circGNB1 with IGF2BP3 was investigated by the Pierce Magnetic RNA Protein pull-down Kit (Thermo Fisher Scientific) in accordance with the manufacturer’s guidelines. Briefly, biotin-labeled circGNB1 or anti-sense RNA were incubated with GSCs at room temperature. After hybridization, the magnetic beads were added to the binding reaction mixture to obtain a probe-magnetic bead complex. Finally, the complexes were washed, boiled and determined by western blotting.

### RNA stability detection

The denovo RNA formation of GSCs was blocked by actinomycin D (Sigma-Aldrich) under condition of 2 μg/ml. Subsequently, total RNA was extracted at 12, 24, 36, 48, and 60 h, which was validated by qRT-PCR in order to check the expression of circGNB1. After treatment of actinomycin D, the circGNB1 half-life as 50% RNA levels can be calculated.

### Xenograft experiments

All the animal studies were performed strictly in accordance with the Animal Care Committee of China Medical University under specific pathogen-free conditions. The ethics number was provided by Institutional Animal Care and Use Committee (IACUC). IACUC Issue No. 2021052. Female BALB/c nude mice (Beijing Vital River Laboratory Animal Technology, Beijing, China) that aged 6 weeks were randomly classified into indicated groups (n = 10, per group). Transfected GSCs (5 × 10^4^ cells per mouse) were subcutaneously injected into the right anterior side of each mouse through a stereotactic apparatus. After orthotopic xenograft, all mice were housed in a breeding colony and maintained on a 12-h light/12-h dark cycle in standard cages with ab libitum access to food and water. All mice were observed daily for signs of distress or death. We used hematoxylin and eosin staining to determine the locations from beginning to the end of each tumor-bearing brain section. After careful comparisons, the section with the largest tumor cross-sectional area for each xenograft was used for the measurement. Tumor volume was calculated according to the formula: V = (L × W^2^) (V = tumor volume, L = the longest diameter of tumor, and W = the shortest diameter of tumor). Survival analysis was calculated by Kaplan–Meier curve.

### Bioinformatics analysis

The circRNA expression in gliomas can be acquired by Gene Expression Omnibus (GEO) datasets. Based on the Cancer Genome Atlas (TCGA, http://cancergenome.nih.gov) and the Chinese Glioma Genome Atlas (CGGA, http://www.cgga.org.cn) dataset, the XPR1 expression ranked from the low to the high, WHO grades, clinical information, and data of molecular biomarkers could be accessed from the publications above. Then, Gene-set enrichment analysis (GSEA, http://www.broadinstitute.org/gsea/index.jsp) was used to analyze any the enrichment of signaling pathways between the high and low XPR1 expression. Besides, Starbase (http://starbase.sysu.edu.cn), TargetScan (www.targetscan.org), miRwalk (mirwalk.umm.uni-heidelberg.de), and miRDB (http://mirdb.org) were used to predict the possible miRNAs targeting XPR1. Then, take advantage of Starbase and circBase (www.circbase.org) to predict potential circRNAs as sponges of miRNA, while Starbase and RBPmap (rbpmap.technion.ac.il) database was used to forecast the proteins binding to circRNAs.

### Statistical analysis

Statistical analysis was processed using SPSS 22.0 software (SPSS, Chicago, IL, USA). All experiments were repeated at least three times, and the results were presented as the mean ± SD. Adopt the two-tailed Student’s t-test, chi-square test, and one-way variance analysis to access differences in variables between groups. For the assessment of the survival rate of each group, the Kaplan–Meier analysis and log-rank test were performed. Two-tailed *P* values < 0.05 were considered statistically significant.

## Results

### CircGNB1 is upregulated in glioma tissues and correlated with poor prognosis.

**Fig. 1:**
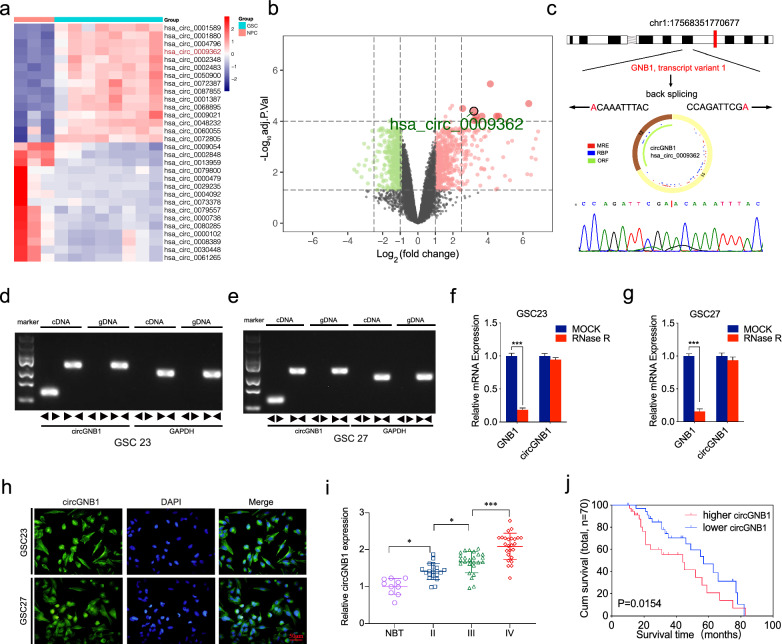
CircGNB1 is up regulated in glioma tissues and correlated with poor prognosis. **a** CircRNA sequences between glioma tissues and normal brain tissues in GSE146463. **b** Volcano plots of circRNAs showed different expression between glioma tissues and NBTs.** c** Schematic illustration of genomic location and back splicing of circGNB1 with the junction sites via Sanger sequencing. **d**,** e** Agarose gel electrophoresis analysis confirmed the presence of circGNB1 in GSCs. **f**,** g** RNase treatment was used to evaluate the stability of circGNB1 and GNB1 mRNA in GSCs. **h** FISH assays confirmed the cellular localization of circGNB1 in GSCs. **i** PCR measured the relative expression of circGNB1 in NBTs (n = 10), grade II (n = 20), grade III (n = 25), and grade IV (n = 25) glioma tissues, and the results showed that circGNB1 was positively associated with the degree of malignancy. **j** Survival curve of glioma patients showed that the survival time was negatively associated with the expression of circGNB1. All data were expressed as mean ± SD, and each experiment was performed in triplicate. *p < 0.05; **p < 0.01; ***p < 0.001

Based on circBase, there are 60 circRNAs originated from GNB1 gene and we detected their expression profiles via GSE146463. Compared with the neural progenitor cells, hsa_circ_0009362 was one of the most significantly up-regulated circRNAs in GSCs (Additional file 6: Table S3, Fig. 1a, b). The splice junction was shown in Fig. 1c. We identified that circGNB1 was transcribed from the GNB1 gene locus, situated at chr1, and formed by back-splicing between the eleventh and the twelfth exons. The junction sites of circGNB1 were CCAGATTCGA and ACAAATTTAC, which were further validated by Sanger sequencing (Fig. 1c). Subsequently, agarose gel electrophoresis assays were performed to detect the expression level of GNB1 in cDNA and genomic DNA (gDNA). Divergent and convergent primers detected the presence of circGNB1 in cDNA while no products were detected in gDNA, which showed that circGNB1 could only be amplified from cDNA (Fig. 1d, e). CircRNAs are resistant to RNase R treatment, whereas linear RNAs could be degraded by RNase R. Thus, RNase R assay was applied to detect the stability of circGNB1. Our results showed that circGNB1 was able to resist digestion caused by RNase R, whereas the linear form of GNB1 was remarkably digested (Fig. 1f, g). Moreover, we further detected the location and expression of circGNB1 by FISH and confirmed that circGNB1 was localized in the cytoplasm (Fig. 1h).

Meanwhile, we found that the expression of circGNB1 was correlated with molecular subtypes of glioma (Table 1). Expression of circGNB1 in glioma tissues was significantly higher than in normal tissues, and circGNB1 in high grade glioma was obviously up-regulated compared with low-grade glioma (Fig. 1i). The survival curve revealed that the higher circGNB1 expressed, the shorter survival time lasted (Fig. 1j). Altogether, these results suggested that circGNB1 was up-regulated in glioma tissues and correlated with poor prognosis, which indicated that circGNB1 might play an essential role in promoting glioma malignancy.

### CircGNB1 promotes the malignant phenotype of GSCs in vitro

We isolated and cultured primary GSCs derived from GBM patients to validate the stemness and differentiation capacity of them. We performed immunofluorescence staining assays and confirmed the presence of stem cell markers, CD133 and nestin in isolated neurospheres (Additional file 1: Fig. S1a). And the multiple fields of neurospheres were showed under a light microscope (Additional file 1: Fig. S1b). Furtherly, we detected and confirmed the differentiation capacity of GSCs using differentiation markers, GFAP and β-III tubulin (Additional file 1: Fig. S1c). The appearance of neurospheres after differentiation for a period was observed under a light microscope (Additional file 1: Fig. S1d).

**Fig. 2:**
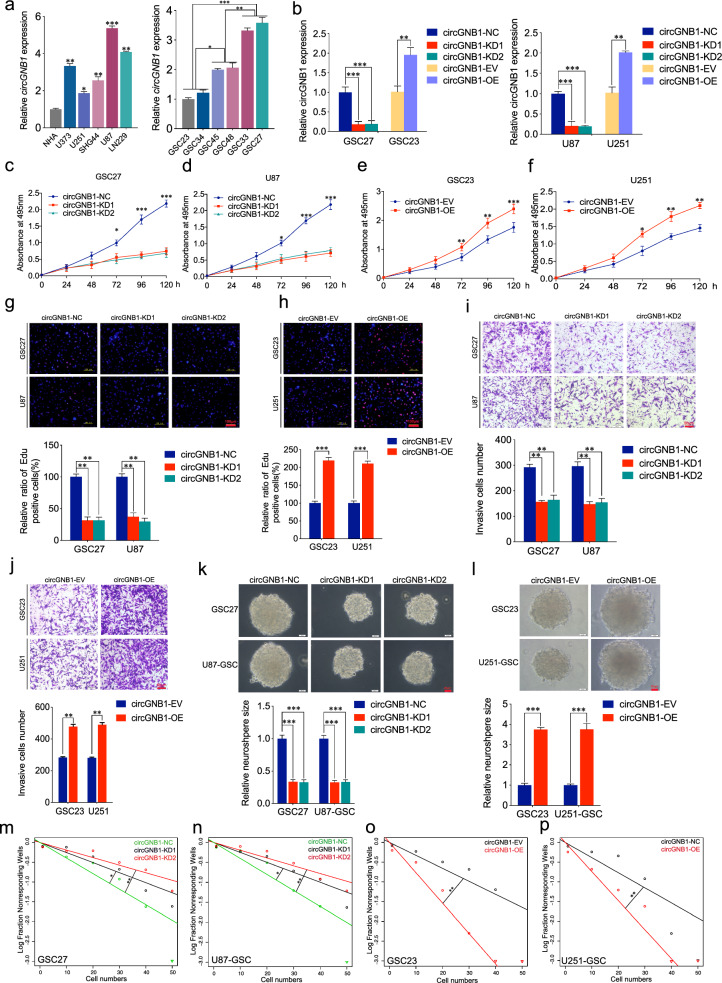
Knockdown of circGNB1 suppressed the malignant phenotype of GSCs in vitro. **a** QRT-PCR assays showed the expression of circGNB1 in different GBM and GSC cell lines. **b** QRT-PCR assays were performed to detect the expression of circGNB1 after knockdown or overexpression treatment by using the lentiviral vector. **c–f** MTS assays showed that the cell viabilities of GSC27 and U87 were significantly decreased after circGNB1 knockdown, while the opposite results could be obtained following circGNB1 overexpression treatment in GSC23 and U251. **g**, **h** Cell proliferation analysis by Edu assays showed that circGNB1 knockdown could reduce the positive rates of GSC27 and U87, whereas circGNB1 overexpression could promote the proliferation of GSC23 and U251. Scale bar = 100 µm. **i**,** j** Transwell assays were performed to detect cell invasion capability using GSC27 and U87 with circGNB1 knockdown or using GSC23 and U251 with circGNB1 overexpression. Scale bar = 50 µm. **k**,** l** The self-renewing abilities of GSCs were measured by neurosphere formation assays with circGNB1 knockdown or circGNB1 overexpression treatment. Scale bar = 20 µm. **m–p** Limiting dilution assays showed circGNB1 knockdown or overexpression affected the neurosphere-forming capacity of GSCs. Data represent mean ± SD (three independent experiments). *p < 0.05; **p < 0.01; ***p < 0.001

The early and rapid progression of GBM is correlated to many significant causes, such as abnormal proliferation, high invasiveness and neurosphere formation ability [23]. We detected the expression of circGNB1 in normal human astrocyte (NHA), GBM cell lines (U373, U251, SHG44, U87, and LN229), and GSC cell lines (GSC23, GSC34, GSC45, GSC48, GSC33, and GSC27) via qRT-PCR. The results showed that U87 and GSC27 cell lines exhibited the highest levels of circGNB1, while U251 and GSC23 cell lines exhibited the lowest levels of circGNB1 (Fig. 2a). Therefore, we selected cell lines (U87, GSC27) which highly expressed circGNB1 as knockdown group by lentivirus transfection, while low expression of circGNB1 cell lines (U251, GSC23) were selected as overexpression group. CircGNB1-KD1 and circGNB1-KD2 were established to knockdown the expression of circGNB1, and qRT-PCR results revealed that the expression of circGNB1 was suppressed efficiently after transfection in GSC27 and U87. In contrast, we overexpressed circGNB1 by using the lentiviral vector and the expression of circGNB1 was remarkably enhanced in GSC23 and U251 (Fig. 2b). MTS and Edu staining assays showed that circGNB1 knockdown could reduce the viability of glioma cells in GSC27 and U87 cells (Fig. 2c, d, g). The opposite results were obtained in the overexpression group in GSC23 and U251 cells (Fig. 2e, f, h). Transwell assays showed that the number of invasive cells in circGNB1 knockdown group was less than the normal group, while the opposite results were obtained in the overexpression group (Fig. 2i, j). The neurosphere formation assays and limiting dilution assays demonstrated that circGNB1 knockdown inhibited the neurosphere-forming capacity, while increased in circGNB1 overexpression group (Fig. 2k–p). These results confirmed that circ-GNB1 plays an important role in promoting the malignant phenotype of GSCs in vitro.

### CircGNB1 serves as a sponge for miR-515-5p and miR-582-3p in GBM cells

**Fig. 3:**
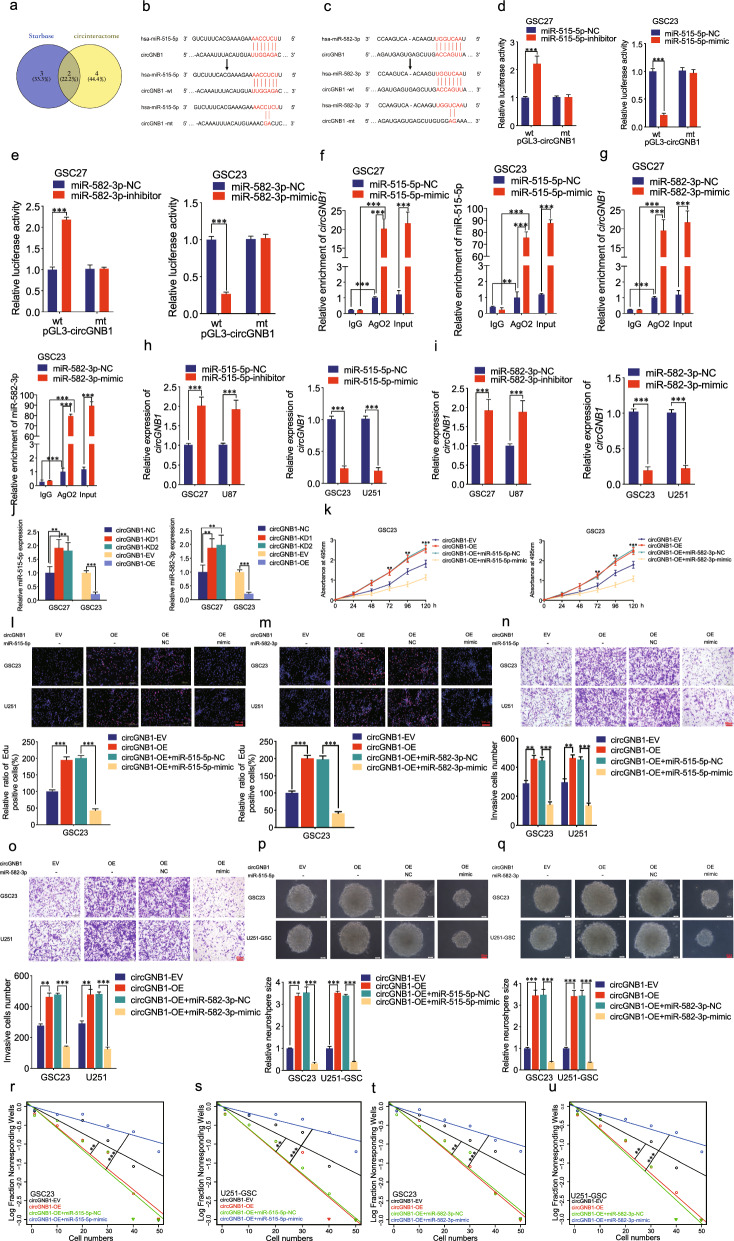
MiR-582-3p and miR-515-5p can bind with circGNB1 and mediated promoting function of circGNB1 on GSCs. **a** Target miRNAs of circGNB1 were deduced by Starbase and circinteractome. **b**, **c** Circular RNA interactome predicted the complementary sequence of circGNB1 as miR-515-5p/miR-582-3p. **d** QRT-PCR analysis showed that the expression of circGNB1 was increased after miR-515-5p/miR-582-3p inhibitor treatment, while decreased following miR-515-5p/miR-582-3p mimic. **e** QRT-PCR showed the expression of miR-515-5p and miR-582-3p after circGNB1 knockdown or overexpression treatment. **f**, **g** The dual luciferase reporter assay was performed in GSC27 and GSC23 co-transfected with reporter plasmid (or the relative mutant reporter of GNB1) and miR-515-5p/miR582-3p inhibitor (or miR-515-5p/miR-582-3p mimic). **h**, **i** The anti-AgO2 RNA immunoprecipitation (RIP) assay was performed after the miR-515-5p/miR-582-3p or negative control was transfected, followed by qPCR to measure the enrichment of circGNB1 or miR-515-5p/miR-582-3p. **j**, **k** MTS assays were performed to detect the cell viabilities of GSC23 after circGNB1 overexpression, the effects of which could be reversed following miR-515-5p/miR-582-3p mimic. **l**, **m** Edu assays showed that the increasing positive rates of GSCs after circGNB1 overexpression could be impaired by miR-515-5p/miR-582-3p mimic. **n**, **o** The transwell assays showed that the enhancing invasion capability after circGNB1 overexpression could be weakened following miR-515-5p/miR-582-3p mimic. **p**, **q** The neurosphere formation assays revealed circGNB1 overexpression could increase the size of the neurospheres of GSCs, whereas reversed by miR-515-5p/miR-582-3p mimic. **r**–**u** The limiting dilution assays demonstrated that circGNB1 overexpression promoted neurosphere-forming capacity of GSCs, which could be repressed following miR-515-5p/miR-582-3p mimic. Data represent mean ± SD (three independent experiments). *p < 0.05; **p < 0.01; ***p < 0.001

To explore whether circGNB1 functions as miRNA sponges, we predicted potential miRNAs which could interact with circGNB1 by analyzing two online databases (Starbase and circinteractome). After taking the intersection with a diagram, we selected two miRNAs (hsa-miR-515-5p, hsa-miR-582-3p) as the miRNA targets of circGNB1 (Fig. 3a). The bioinformatics assay according to Starbase indicated the exact sites at which circGNB1 can bind to miR-515-5p and miR-582-3p (Fig. 3b, c). This analysis result could be confirmed through a dual-luciferase reporter assay experiment. In the experiment, we constructed luciferase reporter plasmids with wild-type (wt) and mutant-type (mt) circGNB1, together with miR-515-5p or miR-582-3p inhibitors and mimics (or their negative control miR-NC). Our results showed that miR-515-5p or miR-582-3p mimics dramatically weakened the luciferase reporter activity of circGNB1-wt instead of circGNB1-mut, whereas miR-515-5p or miR-582-3p inhibitors significantly promoted the activity of circGNB1-wt but not circGNB1-mut (Fig. 3d, e). Additionally, compared with the IgG group, the anti-AgO2 RIP assay indicated that both circGNB1 and miR-515-5p or miR-582-3p were efficiently pulled down by anti-AgO2 antibody. To further understand this molecular mechanism of regulation, the relative enrichment of circGNB1 and miR-515-5p as well as miR-582-3p were much more obvious after miR-515-5p or miR-582-3p mimics treatment, compared with their own negative control groups (Fig. 3f, g). Furthermore, the results in both qRT-PCR experimental groups revealed that the expression of circGNB1 was elevated in miR-515-5p or miR-582-3p inhibitor-treated GSC27 and U87, while decreased in miR-515-5p or miR-582-3p mimic-treated GSC23 and U251 cells (Fig. 3h, i). Besides, qRT-PCR assays suggested that the expression of miR-515-5p and miR-582-3p increased in circGNB1-silenced GSC27 but decreased in circGNB1-overexpressed GSC23 (Fig. 3j).

### MiR-515-5p and miR-582-3p inhibit circGNB1-induced cell proliferation, cell invasion and neurosphere formation in vitro

Then, we performed rescue experiments using miR-515-5p or miR-582-3p mimics to confirm the effects on GSCs proliferation via MTS, Edu and transwell assays. Compared with negative control groups, we observed that cell viability, the rates of Edu-positive GSCs and invasion ability of GSCs were decreased after miR-515-5p/miR-582-3p mimics treatment, which reversed the facilitating effects caused by circGNB1 overexpression (Fig. 3k–o). Moreover, the results in both neurosphere formation and limiting dilution assays showed that neurosphere formation abilities were significantly promoted in circGNB1-overexpressed GSC23 and U251-GSC cells, while the opposite results were achieved following treatment of miR-515-5p or miR-582-3p mimics (Fig. 3p–u). In conclusion, all these data implied that circGNB1 regulated the malignant phenotype in glioma cells by acting as a sponge for miR-515-5p and miR-582-3p.

### The 3′-UTR of XPR1 mRNA is the common direct target of miR-515-5p and miR-582-3p

**Fig. 4:**
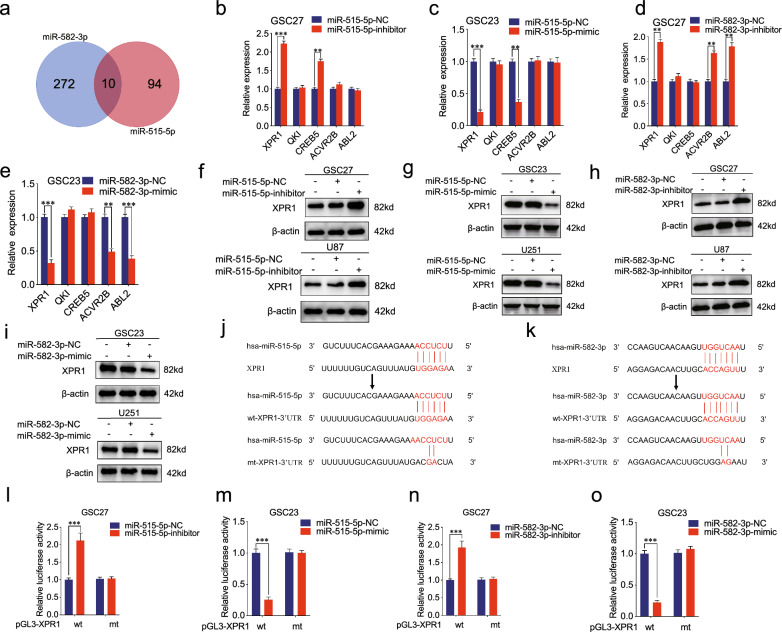
The 3′UTR of XPR1 mRNA is the common direct target of miR-515-5p and miR-582-3p. **a** Identification and intersection of a mRNA potentially targeted with both miR-515-5p and miR-582-3p based on microRNA, miRWalk, Starbase, and TargetScan databases. **b**–**e** qPCR showed the relative gene expression in GSCs after miR-515-5p/miR-582-3p inhibitor (or mimic) treatment. **f**–**i** Western blotting showed the expression of XPR1 following miR-515-5p/miR-582-3p inhibitor (or mimic) treatment. **j**, **k** Schematic diagram of the putative miR-515-5p and miR-582-3p binding site in XPR1. **l**–**o** The luciferase reporter assays showed that miR-515-5p/miR-582-3p inhibitor (or mimic) affected the 3′UTR region of XPR1 mRNA in GSCs. Data represent mean ± SD (three independent experiments). *p < 0.05; **p < 0.01; ***p < 0.001

To further explore the target gene potentially binding to both miR-515-5p and miR-582-3p, we made an identification and intersection based on microRNA, Starbase, miRDB and TargetScan databases. Finally, we found 10 potential common candidate genes, including QKI, ABL2, XPR1, ACVR2B, CREB5, TMEM33, UBE2H, ATAB2D, ZBTB44 and STRN (Fig. 4a). Then we searched related studies to figure out respective functions. We found that QKI could inhibit glioma stem cell stemness [24]. And ABL2 could suppress cancer progression in prostate cancer, cervical carcinoma and glioma [25, 26], whereas promoting hepatocellular carcinomas and gastric cancer progression [27, 28]. In addition, previous studies revealed that XPR1, ACVR2B and CREB5 could promote progression of diverse cancers, but they were not reported in glioma [18, 29, 30]. The other five genes (TMEM33, UBE2H, ATAB2D, ZBT44 and STRN) were not reported in cancers. In order to figure out the oncogene associated with glioma, we selected five genes to preform qRT-PCR experiments, including XPR1, QKI, CREB5, ACVR2B and ABL2. Interestingly, the results revealed that the expression of XPR1 and CREB5 was increased when treated by miR-515-5p inhibitor in GSC27, whereas decreased following miR-515-5p mimic treatment in GSC23 (Fig. 4b, c). The expression of XPR1, ACVR2B and ABL2 was increased after miR-582-3p inhibitor treatment in GSC27, while restrained following miR-582-3p mimic treatment in GSC23 (Fig. 4d, e). Taking the intersection between them, we presumed that XPR1 was the candidate targeted gene between miR-515-5p and miR-582-3p in glioma.

Our results in western blotting showed that both miR-515-5p and miR-582-3p inhibitors could upregulate XPR1 expression in GSC27 and U87, whereas the mimics of miR-515-5p and miR-582-3p could downregulate XPR1 expression in GSC23 and U251 (Fig. 4f–i). Besides, StarBase prediction analysis suggested that miR-515-5p and miR-582-3p could co-target the 3′-UTR region of XPR1 gene and thus we designed luciferase report assays (Fig. 4j, k). And the luciferase reporter assays revealed that miR-515-5p and miR-582-3p mimics remarkably repressed the relative activity of the GSC23 cell group transfected with wild-type (wt) GNB1 luciferase reporter vector. But it could not function as well in group transfected with mutant-type (mut) (Fig. 4m, o). Furthermore, the opposite results could be obtained when the GSC27 cell group was treated following miR-515-5p and miR-582-3p inhibitors (Fig. 4i, n). In conclusion, the above results indicated miR-515-5p and miR-582-3p could bind with 3′-UTR of XPR1 to regulate its expression.

### CircGNB1 mediated the malignant phenotype of GSCs by acting as ceRNA of miR-515-5p and miR-582-3p to regulate XPR1 expression

**Fig. 5:**
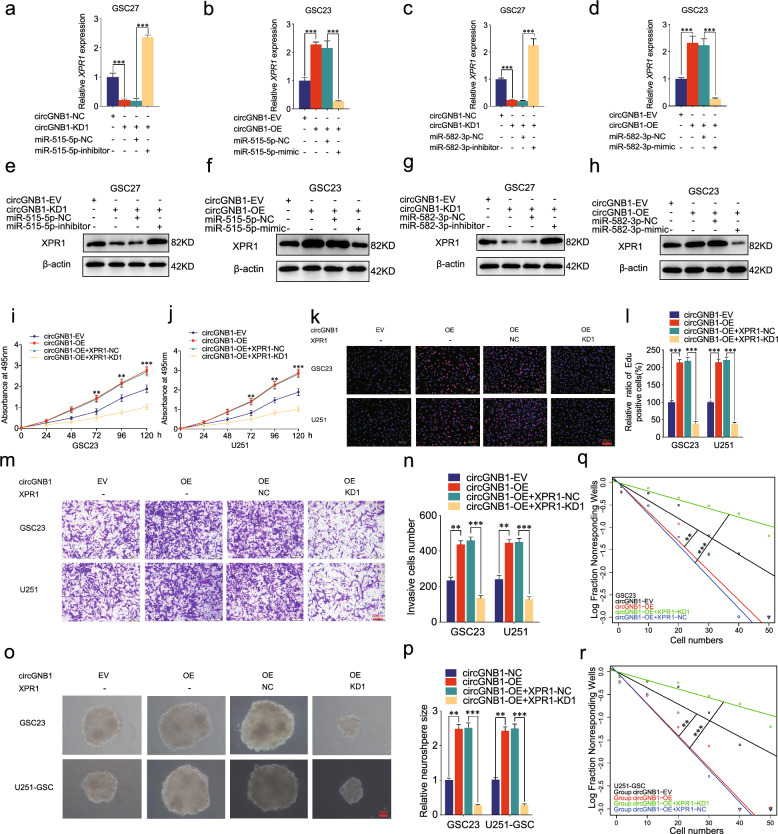
CircGNB1 mediated the malignant phenotype of GSCs by acting as ceRNA of miR-515-5p and miR-582-3p to regulate XPR1 expression. **a**–**d** qPCR assays showed miR-515-5p/miR-582-3p inhibitor could reverse the expression of XPR1 reduced by circGNB1 knockdown, while miR-515-5p/miR-582-3p mimic could subside the expression of XPR1 promoted by circGNB1 overexpression in GSCs. **e**–**h** Western blotting revealed the effects of miR-515-5p/miR-582-3p inhibitor (or mimic) on XPR1 protein expression. **i**, **j** MTS assays showed XPR1 knockdown reversed the cell viabilities promoted by circGNB1 overexpression. **k**, **l** Edu assays showed that XPR1 knockdown reversed the positive rates of GSC23 and U251 caused by circGNB1 overexpression. **m**, **n** The transwell assays revealed that XPR1 knockdown could reverse the invasion capabilities enhanced by circGNB1 overexpression. **o**, **p** The neurosphere formation assays demonstrated that XPR1 knockdown could reverse the size of neurosphere promoted by circGNB1 overexpression. **q**, **r** The limiting dilution assays showed that XPR1 knockdown affected the neurosphere-forming capacity of GSCs with circGNB1 overexpression. Data represent mean ± SD (three independent experiments). *p < 0.05; **p < 0.01; ***p < 0.001

Subsequently, we performed corresponding rescue experiments using miR-515-5p or miR-582-3p inhibitors after silencing circGNB1, and using miR-515-5p or miR-582-3p mimics following overexpressing circGNB1. In comparison with the negative control group, qRT-PCR results suggested that downregulating circGNB1 inhibited the XPR1 mRNA expression in GSC27. Moreover, miR-515-5p and miR-582-3p inhibitors treatment could reverse the suppressing effects caused by circGNB1 knockdown (Fig. 5a, c). On the contrary, miR-515-5p and miR-582-3p mimics abated the improving expression of XPR1 when circGNB1 was overexpressed (Fig. 5b, d). Besides, western blotting showed that the corresponding decrease in XPR1 protein levels suppressed by circGNB1 knockdown could be counteracted and upregulate by miR-515-5p or miR-582-3p inhibitor in GSC27 (Fig. 5e, g). Simultaneously, the opposite results could be obtained in circGNB1 overexpressed GSC23 following miR-515-5p or miR-582-3p mimic treatment (Fig. 5f, h). In order to further investigate how circGNB1 promoted GSCs viability, proliferation ability, invasion ability and self-renewing capacity, we performed MTS, Edu, transwell, neurosphere formation and limiting dilution rescue assays using XPR1 knockdown treatment. It could be confirmed that downregulating XPR1 expression could counteract the GSCs viability, proliferative potential invasion ability and self-renewing capacity strengthened by overexpressing circGNB1 (Fig. 5i–r). In summary, circGNB1 promoted the malignant phenotype of GSCs via sponging miR-515-5p and miR-582-3p to regulate XPR1 expression.

### XPR1 promote the malignant phenotype of GSCs via IL6/JAK2/STAT3 signaling axis

**Fig. 6:**
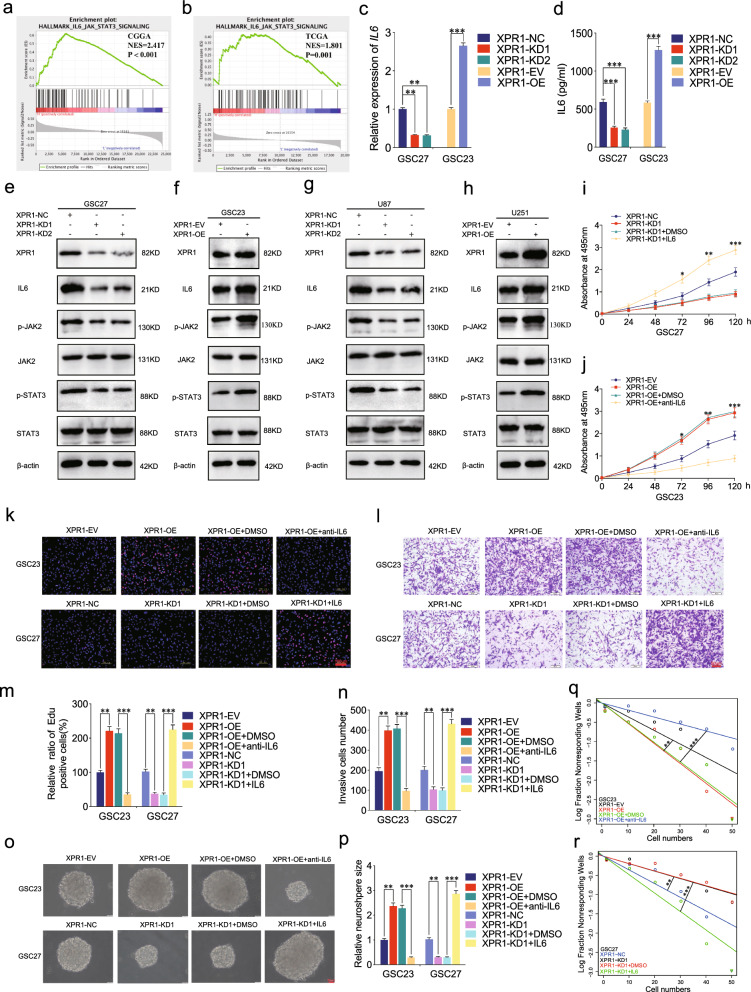
XPR1 promote the malignant phenotype of GSCs via IL6/JAK2/STAT3 signaling axis. **a**, **b** CGGA and TCGA datasets showed that the expression of XPR1 was positively related to enrichment of IL6-mediate JAK2/STAT3 signaling. **c**–**h** qRT-PCR (**c**), ELISA (**d**), and western blotting (**e**–**h**) revealed that XPR1 could alter the expression of IL6 via JAK2/STAT3 axis in GSCs. **i**–**n** MTS (**i**, **j**), Edu (**k**, **m**) and transwell (**l**, **n**) assays indicated that IL6 could reverse the cell viabilities, proliferation abilities, and invasion capabilities repressed by XPR1 knockdown in GSC27, whereas anti-IL6 could reverse the cell viabilities, proliferation abilities, and invasion capabilities promoted by XPR1 overexpression in GSC23; **o**–**r** The neurosphere formation (**o**, **p**) and limiting dilution assays (**q**, **r**) demonstrated that IL6 could reverse the size of neurosphere repressed by XPR1 knockdown, whereas anti-IL6 could reverse the neurosphere-forming capacity promoted by XPR1 overexpression. Data represent mean ± SD (three independent experiments). *p < 0.05; **p < 0.01; ***p < 0.001

To further verify the underlying molecular mechanism of how XPR1 facilitated malignant progression on GSCs, we performed GSEA on CGGA and TCGA datasets toward XPR1 expression. The results in both datasets demonstrated that XPR1 expression was positively associated with enrichment of IL6-mediated JAK2/STAT3 signaling pathway (Fig. 6a, b). Subsequently, our results in qRT-PCR and ELISA assays revealed that the expression of IL6 after XPR1 knockdown treatment was much less than that of control group, whereas the opposite results were obtained after XPR1 overexpression (Fig. 6c, d). Then we performed western blotting and found that XPR1 knockdown could significantly downregulate XPR1 expression as well as downstream molecules protein levels, involving IL6, p-JAK2 and p-STAT3 in JAK2/STAT3 signaling pathway in GSC27 and U87 (Fig. 6e, g). In contrast, the opposite results could be obtained following XPR1 overexpression treatment in GSC23 and U251 (Fig. 6f, h). Additionally, we performed functional rescue experiments using human recombinant IL6 and anti-IL6 treatment to antagonize IL6. The results in MTS, Edu, transwell and neurosphere formation assays demonstrated that cell viability, the rates of Edu-positive GSCs, invasion capacity and self-renewing capacity were dramatically decreased in XPR1 knockdown GSC27, whereas reversed after additional IL6 treatment. The opposite results could be obtained following XPR1 overexpression, which also reversed after anti-IL6 treatment (Fig. 6i–r). Collectively, these results indicated that XPR1 promoted the malignant phenotype of GSCs via IL6/JAK2/STAT3 signaling pathway.

Moreover, we detected the influence of circGNB1 on IL6/JAK2/STAT3 pathway. The results in qRT-PCR, ELISA, and western blotting assays showed that circGNB1 could promote the IL-6 expression, while the opposite results could be obtained after anti-IL6 treatment (Additional file 2: Fig. S2a–c). And Edu, transwell, neurophere formation assays revealed that circGNB1 could promote the rates of Edu-positive GSCs, invasion capacity and neurophere size, while anti-IL6 treatment could reverse the results (Additional file 2: Fig. S2d–j). In summary, our results revealed that circGNB1 could promote the malignant phenotype of GSCs via IL6/Jak2/STAT3 pathway.

### IGF2BP3 can bind to and maintain the stability of circGNB1 and IGF2BP3 can mediate the promoting effects of circGNB1 on GSCs

**Fig. 7:**
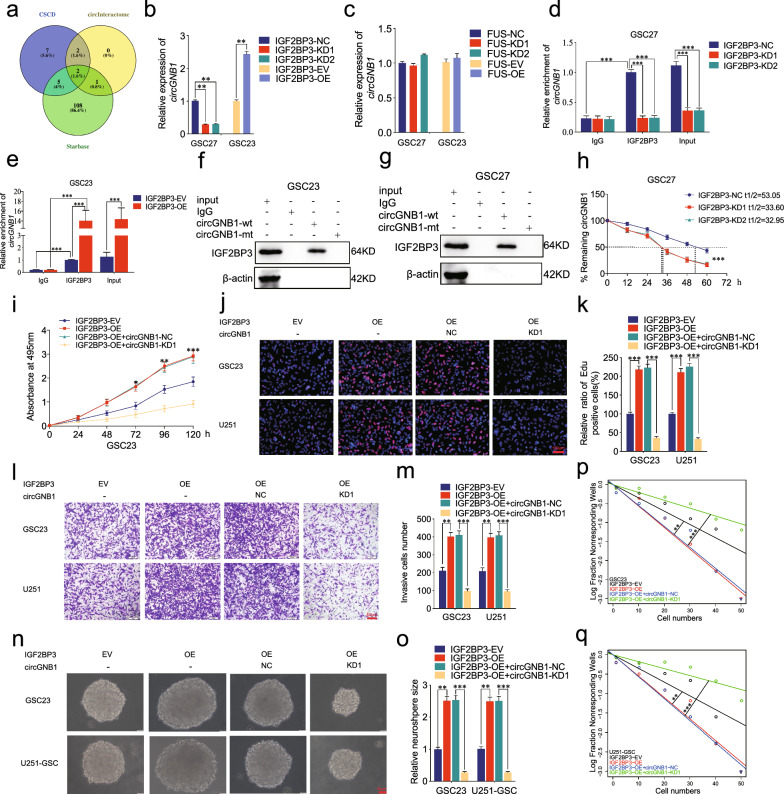
IGF2BP3 can bind to and maintain the stability of circGNB1 and IGF2BP3 can mediate the promoting effects of circGNB1 on GSCs. **a** Identification and intersection of RBPs (IGF2BP3, FUS) potentially bound to circGNB1 based on CSCD, circInteractome, and Starbase. **b**, **c** qRT-PCR showed that IGF2BP3 knockdown or overexpression significantly affected the expression of circGNB1, whereas FUS knockdown or overexpression couldn’t. **d**, **e** The RIP assay was performed after IGF2BP3 knockdown or overexpression, followed by qRT-PCR to detect the enrichment of circGNB1. **f**, **g** The RNA pull-down assays showed IGF2BP3 protein immunoprecipitation with circGNB1 as detected by western blotting. **h** qRT-PCR assay was performed to detect the relative expression levels of circGNB1 treated with actinomycin D at different time points in IGF2BP3 knockdown GSC27. **i**–**m** MTS (**i**), Edu (**j**, **k**), and transwell (**l**, **m**) assays revealed circGNB1 knockdown could reverse the cell viabilities, proliferation capacities, and invasion abilities promoted by IGF2BP3 overexpression. **n**–**q** The neurosphere formation (**n**, **o**) and limiting dilution assays (**p**, **q**) showed circGNB1 knockdown could reverse the neurosphere formation facilitated by IGF2BP3 overexpression. Data represent mean ± SD (three independent experiments). *p < 0.05; **p < 0.01; ***p < 0.001

RBPs are involved in the interaction and regulation of RNAs and contribute to tumor cells biology process [31]. To furtherly determine whether corresponding RBPs could interact with circGNB1, we predicted that IGF2BP3 and FUS were most likely to regulate its expression according to CSCD, circInteractome and Starbase (Fig. 7a). Subsequently, qRT-PCR was performed to detect the expression of circGNB1, compared with each negative group, in IGF2BP3/FUS knockdown group and IGF2BP3/FUS overexpression group. However, the expression of circGNB1 was remarkably attenuated in GSC27 using IGF2BP3 knockdown, while increased in GSC23 following IGF2BP3 overexpression treatment, respectively (Fig. 7b). But there was no clear difference in FUS group. To investigate whether IGF2BP3 bound to circGNB1, we performed a RIP assay using IGF2BP3 knockdown and IGF2BP3 overexpression treatment. The results showed that the enrichment of circGNB1 in the anti-IGF2BP3 group was significantly increased in comparison with that in the IgG treated group. IGF2BP3 knockdown decreased the enrichment of circGNB1 in GSC27, whereas IGF2BP3 overexpression further increased the enrichment of circGNB1 in GSC23 (Fig. 7c, d, e). In addition, RNA pull-down assays indicated that biotinylated circGNB1-wt could pull down IGF2BP3 in both GSC23 and GSC27, while circGNB1-mt could not (Fig. 7f, g). Next, RNA stability assays demonstrated that the half-life of circGNB1 was significantly shortened following IGF2BP3 knockdown compared with negative control group (Fig. 7h). Moreover, we performed rescue phenotype assays using circGNB1 knockdown to explore the relative effects of IGF2BP3 on GSCs. The circGNB1 knockdown treatment could counteract the facilitating effects of GSCs viability (Fig. 7i), Edu positive rates (Fig. 7j, k), invasion (Fig. 7l, m) and neurosphere formation abilities (Fig. 7n–q) caused by IGF2BP3 overexpression in GSC23 and U251. Altogether, these results above showed that IGF2BP3 could bind to and maintain the stability of circGNB1, thus mediating the promoting effects of circGNB1 on GSCs.

### CircGNB1 promoted GSCs tumorigenesis in vivo

**Fig. 8:**
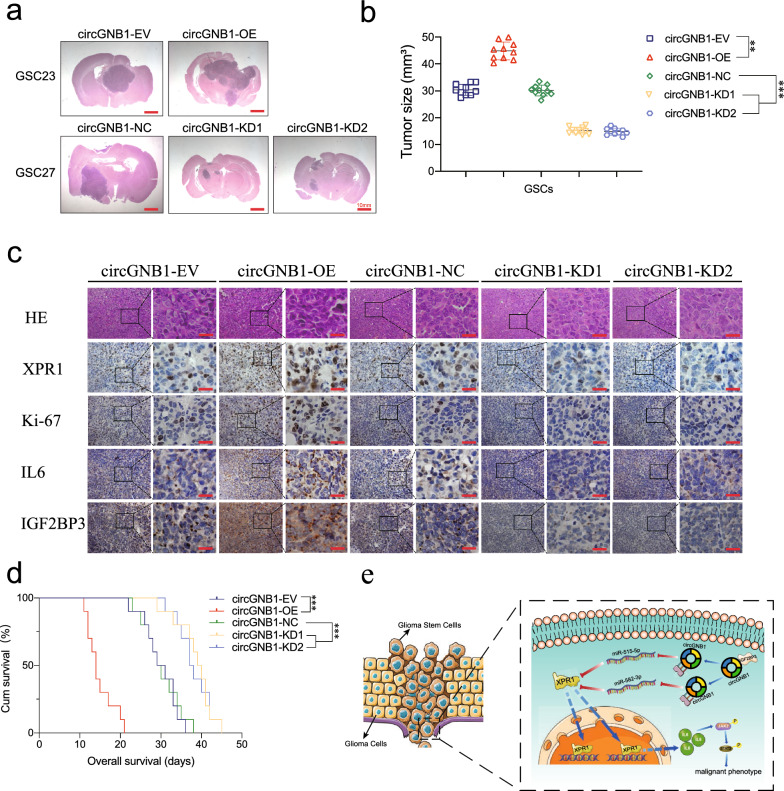
CircGNB1 promoted GSCs tumorigenesis in vivo. **a** Hematoxylin and eosin staining of intracranial tumor plantation showed the tumor size in the coronal location of five groups. Scale bar = 10 mm. **b** The measured tumor volumes among each group are indicated. **c** Representative immunohistochemical staining showed the changes in XPR1, Ki-67, IL6, and IGF2BP3 in each orthotopic xenograft model. Scale bar = 50 µm. **d** Kaplan–Meier survival curves revealed nude mice’s survival times in each group (n = 10). **e** Schematic diagram demonstrated that IGF2BP3/circGNB1/miR-515-5p/miR-582-3p/XPR1 promoted malignant phenotype of GSCs via IL6/JAK2/STAT3 axis. Data represent mean ± SD (three independent experiments). *p < 0.05; **p < 0.01; ***p < 0.001

After demonstrating the effects of circGNB1 in vitro, we performed orthotopic xenografts to further investigate the role of circGNB1 in glioma tumorigenesis in vivo. Excised tumor area of each mouse was calculated, and we found that the tumor volume in circGNB1-overexpressed mice was significantly larger than that in negative control group. The opposite result could be obtained after circGNB1-knockdown treatment (Fig. 8a, b). Next, we performed immunohistochemistry assays to detect the effects of IGF2BP3/circGNB1/miR-515-5p/miR-582-3p/XPR1/IL6 axis on tumor tissues. Consistent with the cytology results, we found that circGNB1 overexpression could promoted the expression of XPR1, Ki67, IL6, and IGF2BP3 in vivo, whereas the opposite results could be obtained following circGNB1 knockdown treatment (Fig. 8c). Meanwhile, we calculated the survival analysis by Kaplan–Meier curve, and the results showed that the survival time of circGNB1-overexpressed group was much shortened than that in negative control group. On the contrary, the mice in circGNB1-knockdown group survived longer than negative group (Fig. 8d). Further, we drew the schematic diagram to illustrate our conclusion (Fig. 8e). Collectively, our results suggested that circGNB1 was critical for glioma tumorigenicity via above signaling axis.

## Discussion

Recently, emerging evidence has confirmed that circRNAs play an oncogene role in various types of tumors, such as pancreatic cancer, breast cancer, bladder cancer, glioma and so on [11, 32–34]. CircRNAs are covalently closed RNA rings without poly-adenylated tails, and they are wildly expressed in eukaryotes [6]. Abnormalities in circRNA expression significantly impact the tumorigenesis and biological behaviors. Recent studies show that circRNAs positively regulate glioma in proliferation, invasion, angiogenesis, resistance and so on. For example, previous study showed that circ_0072083 and circ_0110757 could enhance temozolomide resistance in glioma [35, 36]. Besides, circRNA could also promote tumorigenesis in glioma via p53 [37, 38]. Circ-DICER1 could regulate the angiogenesis of glioma via sponging miRNAs [39]. Nevertheless, the function of many novel circRNAs still remains unknown in glioma. We found that the expression of circGNB1 in glioma tissues was significantly higher than the NBTs, which indicated circGNB1 might be an important biomarker for the diagnosis, prognosis, and treatment of glioma. High expression of circGNB1 in high-grade glioma is closely related to poor patient survival. Therefore, to elucidate the molecular mechanism and function of circGNB1 in glioma appeared to be especially essential. CircGNB1 may play a vital role on glioma progression as a potential cancer biomarker.

Previous research confirmed that miRNAs could function by binding to 3′UTR of transcription factors of downstream genes [40]. We predicted the existence of binding sites between circGNB1 and miR-515-5p/miR-582-3p through bioinformatics analysis, then dual-luciferase reporter assay confirmed the conjunction. Previous studies showed that miR-515-5p and miR-582-3p played vital roles in tumor progression. For example, it was reported that miR-515-5p could be inhibited by LINC00673, which promoted the proliferation of breast cancer cells [41]. Besides, miR-582-3p could also be sponged by circSHKBP1 to promote gastric cancer progression [42]. The results in our study further demonstrated the important function of miR-515-5p/miR-582-3p in glioma cells, especially in GSCs. In addition, circRNA could biologically play a more prominent role via sponging multiple miRNAs compared with single miRNA. For example, circSERPINE2 could inhibit miR-361-3p and miR-324-5p to promote the development of glioblastoma [43].

We further confirmed that miR-515-5p and miR-582-3p bound to 3′UTR of XPR1 and negatively regulated the expression of XPR1. XPR1 is a known oncogene which plays vital roles in various cancers. For example, XPR1 could promote progression of tongue squamous cell carcinoma via activation of NF-κB signaling [42]. XPR1 could also promote the tumorigenicity of esophageal squamous cell carcinoma [44]. However, the function of XPR1 in glioma remains unclear. To elucidate molecular mechanisms how XPR1 facilitated malignant progression of GSCs, we further performed a series of assays on molecular pathways and verified that XPR1 functioned via IL6/JAK2/STAT3 axis. It was reported that IL6 played an important role in glioma [45]. Our results further confirmed the key role of IL6 in glioma progression.

RBPs play important biological roles in dynamic spatiotemporal regulation, such as RNA splicing, mRNA stability, mRNA localization, and translation [46]. IGF2BP3 is a known oncogenic protein and belongs to a novel family of RBPs (IGF2BPs). Functionally, IGF2BP3 can bind to and maintain the stability of downstream mRNAs to promote cancer progression, such as CDK4 and CDK6 [47, 48]. For example, IGF2BP3 activity could be elevated by circIGHG, thus promoting metastasis of oral squamous cell carcinoma (OSCC) via epithelial-mesenchymal transition [49]. Moreover, it was reported that circNEIL3 could inhibit IGF2BP3 ubiquitination to promote glioma progression [17]. Furthermore, in breast cancer, IGF2BP3 could inhibit miRNA-3614 maturation and protect TRIM25 mRNA from degradation to promote breast cancer cell proliferation [50]. We forecasted the binding site of IGF2BP3 and circGNB1 through bioinformatics database, then the results in RIP assays confirmed that IGF2BP3 promoted and maintained the expression of circGNB1.

Nowadays, numerous studies have proved that circRNAs can regulate the development and progression of various cancers in several manners. For example, circNDUFB2 functions as a scaffold to enhance the interaction between TRIM25 and IGF2BPs, thus facilitating the progression and metastasis of non-small cell lung cancer [51]. CircNEIL3 could be packaged into exosomes and transmitted to infiltrated tumor associated macrophages, enabling them to acquire immunosuppressive properties in glioma progression [17]. Besides, an undescribed secretory E-cadherin protein variant (C-E-Cad) encoded by a circular E-cadherin (circ-E-Cad) RNA could active EGFR signaling in GBM [52]. In addition to the partial regulation of ceRNA mechanism, there may be other downstream factors regulated by circGNB1 to promote the progression in GBM. This warrants further investigation in the future.

Collectively, our results confirmed that IGF2BP3/circGNB1/miR-515-5p/miR-582-3p/XPR1 axis could promote tumorigenesis and malignant progression of glioma via IL6/JAK2/STAT3 signaling pathway.

## Conclusions

In conclusion, our study demonstrates that circGNB1 is significantly upregulated in glioma tissues compared with normal brain tissue. Mechanistically, circGNB1 promotes the malignant phenotype of GSCs via sponging miR-515-5p and miR-582-3p. Furtherly, IGF2BP3 can bind to and maintain the stability of circGNB1, thus enhancing the facilitating effects on GSCs.

## Supplementary Information

**Additional file 1. Fig. S1**: Isolation and validation of patient derived glioma stem cells (GSCs). **a** Immunofluorescence staining of CD133 and nestin in patient-derived GSCs. Scale bar = 20 µm. **b** Multiple fields of neurospheres under a light microscope. Scale bar = 50 µm. **c** Immunofluorescence showed the expression of GFAP and β-III tubulin in GSCs. Scale bar = 20 µm. **d** Differentiation of GSCs under a light microscope. Scale bar = 20 µm.

**Additional file 2. Fig. S2**: CircGNB1 promoted the malignant phenotype of GSCs via IL6/JAK2/STAT3 pathway. **a** QRT-PCR showed that anti-IL6 treatment could reverse the expression of IL6 promoted by circGNB1 overexpression. **b** ELISA assays showed that circGNB1 was positively related to the IL6 expression. **c** Western blotting assays showed that circGNB1 overexpression promoted IL6/JAK2/STAT3 expression, while circGNB1 knockdown inhibited the pathway. **d**–**g** The edu (**d**, **e**) and transwell (**f**, **g**) assays revealed that anti-IL6 treatment could reverse the promoting effect caused by circGNB1 overexpression. **h**–**j** The neurosphere formation assays showed that circGNB1 overexpression promoted the neurosphere formation, while anti-IL6 treatment could inhibited the size of neurosphere.

**Additional file 3. Fig. S3**: The expression of other five target genes after specific treatment. The qRT-PCR experiments showed that the expression of UBE2H was increased after miR-515-5p inhibitor treatment (**a**), whereas reduced by miR-582-3p mimic treatment (**b**). The expression of STRN was increased after miR-582-3p inhibitor treatment (**c**), whereas reduced by miR-582-3p mimic treatment (**d**).

**Additional file 4. Table S1**: SiRNA sequences.

**Additional file 5. Table S2**: PCR primers sequences.

**Additional file 6. Table S3**: The expression of GNB1 originated circRNAs in GSE146463.

## Data Availability

We have obtained consent to publish the article from all the authors.

## Abbreviations

GSCs: Glioma stem cells
circRNA: Circular RNA
miRNA: MicroRNA
RBP: RNA binding protein
IL6: Interleukin-6
3′-UTR: 3′Untranslated region
GSEA: Gene set enrichment analysis
TCGA: The Cancer Genome Atlas
CGGA: Chinese Glioma Genome Atlas
qRT-PCR: Real-Time Quantitative Reverse Transcription PCR
IHC: Immunohistochemistry
ELISA: Enzyme-linked immunosorbent assay
ceRNA: Competitive endogenous RNA
GBM: Glioblastoma
