# Supplementary figures and images for: CircGNB1 facilitates the malignant phenotype of GSCs by regulating miR-515-5p/miR-582-3p-XPR1 axis
Source: Cancer Cell Int. 2023 Jul 5;23:132. doi: 10.1186/s12935-023-02970-2 (PMC10320909; doi:10.1186/s12935-023-02970-2)

a

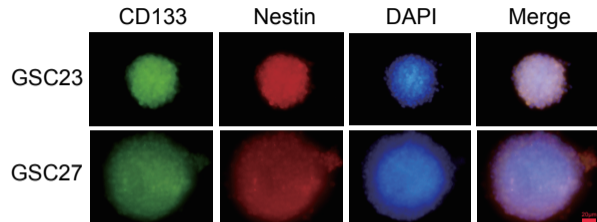

c

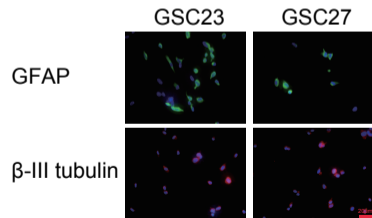

b

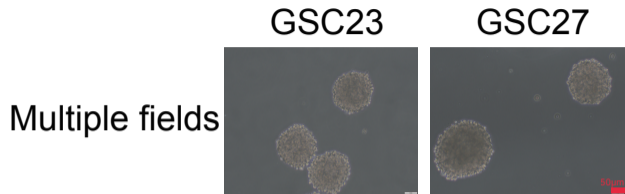

d

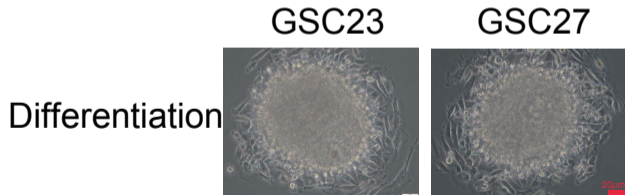

Supplement: Supplementary file 1 — Additional file 1. Fig. S1: Isolation and validation of patient derived glioma stem cells (GSCs). a Immunofluorescence staining of CD133 and nestin in patient-derived GSCs. Scale bar = 20 µm. b Multiple fields of neurospheres under a light microscope. Scale bar = 50 µm. c Immunofluorescence showed the expression of GFAP and β-III tubulin in GSCs. Scale bar = 20 µm. d Differentiation of GSCs under a light microscope. Scale bar = 20 µm. [file 12935_2023_2970_MOESM1_ESM.pdf]

**a**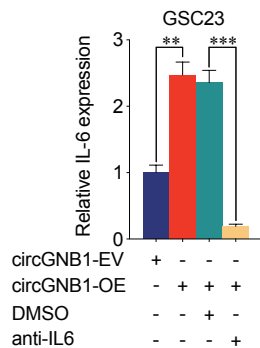**b**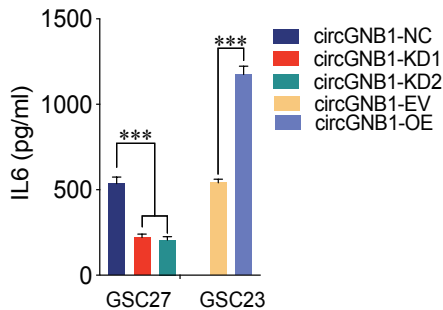**c**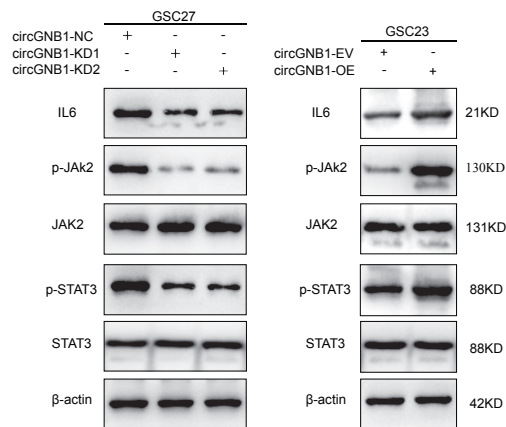**d**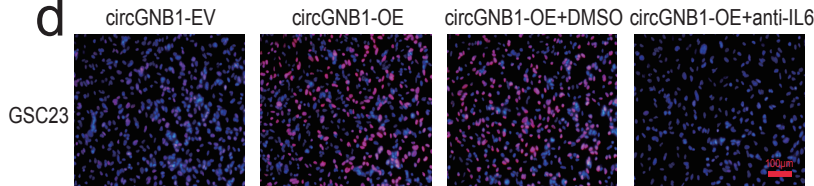**e**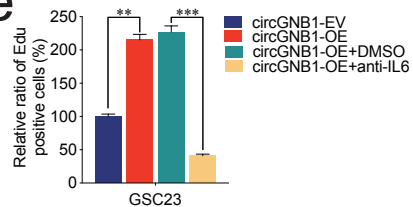**f**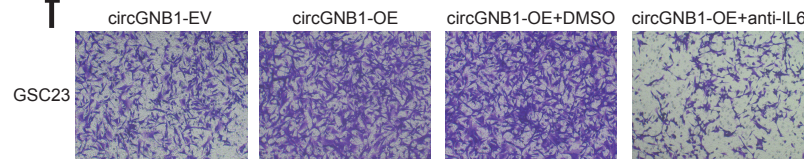**g**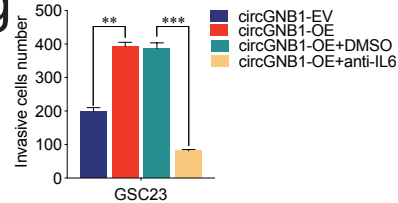**h**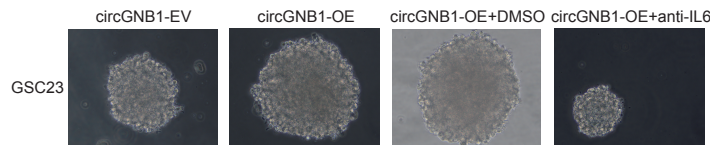**i**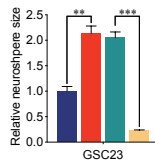**j**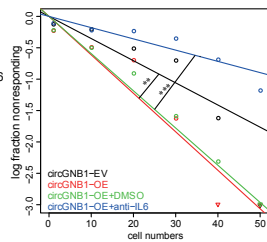

Supplement: Supplementary file 2 — Additional file 2. Fig. S2: CircGNB1 promoted the malignant phenotype of GSCs via IL6/JAK2/STAT3 pathway. a QRT-PCR showed that anti-IL6 treatment could reverse the expression of IL6 promoted by circGNB1 overexpression. b ELISA assays showed that circGNB1 was positively related to the IL6 expression. c Western blotting assays showed that circGNB1 overexpression promoted IL6/JAK2/STAT3 expression, while circGNB1 knockdown inhibited the pathway. d–g The edu (d, e) and transwell (f, g) assays revealed that anti-IL6 treatment could reverse the promoting effect caused by circGNB1 overexpression. h–j The neurosphere formation assays showed that circGNB1 overexpression promoted the neurosphere formation, while anti-IL6 treatment could inhibited the size of neurosphere. [file 12935_2023_2970_MOESM2_ESM.pdf]

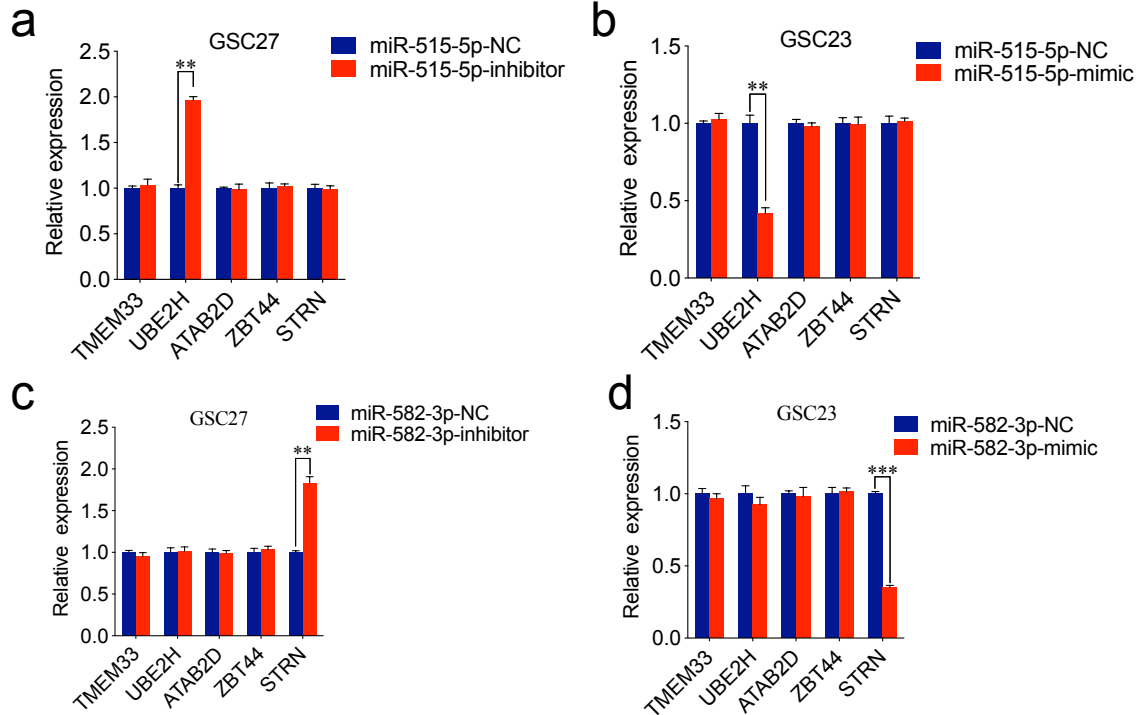

Supplement: Supplementary file 3 — Additional file 3. Fig. S3: The expression of other five target genes after specific treatment. The qRT-PCR experiments showed that the expression of UBE2H was increased after miR-515-5p inhibitor treatment (a), whereas reduced by miR-582-3p mimic treatment (b). The expression of STRN was increased after miR-582-3p inhibitor treatment (c), whereas reduced by miR-582-3p mimic treatment (d). [file 12935_2023_2970_MOESM3_ESM.pdf]
