# Supplementary material for: CircGNB1 facilitates the malignant phenotype of GSCs by regulating miR-515-5p/miR-582-3p-XPR1 axis
Source: Cancer Cell Int. 2023 Jul 5;23:132. doi: 10.1186/s12935-023-02970-2 (PMC10320909; doi:10.1186/s12935-023-02970-2)
Supplement: Supplementary file 4 — Additional file 4. Table S1: SiRNA sequences. [file 12935_2023_2970_MOESM4_ESM.docx]

| **Gene** | **Forward sequences** | **Reverse sequences** |
| --- | --- | --- |
| circGNB1-KD1 | UCUUAGUGCUCUUCAAUGCCA | GCAUUGAAGAGCACUAAGAUC |
| circGNB1-KD2 | AUCUUAGUGCUCUUCAAUGCC | CAUUGAAGAGCACUAAGAUCG |
| XPR1-KD1 | UGAAUAUUCCACAAAAUAGGC | CUAUUUUGUGGAAUAUUCAUU |
| XPR1-KD2 | AGUACAAUGAAUAUUCCACAA | GUGGAAUAUUCAUUGUACUGA |
| IGF2BP3-KD1 | UUUCGUAUCUGAAGUUUCCGA | GGAAACUUCAGAUACGAAAUA |
| IGF2BP3-KD2 | AUUUCGUAUCUGAAGUUUCCG | GAAACUUCAGAUACGAAAUAU |
| siRNA-NC | UUCUUCGAAGGUGUCACGUTT | ACGUGACACCUUCGAAGAATT |

**Supplementary Table 1. siRNA sequences**
