# Supplementary material for: CircGNB1 facilitates the malignant phenotype of GSCs by regulating miR-515-5p/miR-582-3p-XPR1 axis
Source: Cancer Cell Int. 2023 Jul 5;23:132. doi: 10.1186/s12935-023-02970-2 (PMC10320909; doi:10.1186/s12935-023-02970-2)
Supplement: Supplementary file 5 — Additional file 5. Table S2: PCR primers sequences. [file 12935_2023_2970_MOESM5_ESM.docx]

**Supplementary Table 2. PCR Primers sequences**

**RT-qPCR Primers**

| **Gene** | **Forward Primer** | **Reverse Primer** |
| --- | --- | --- |
| circGNB1 | TGGTGTTACTACGCTGCGAC | CACTGAAGGCCAGTTTAAGGTC |
| XPR1 | ACCGACTTTACTCTTCCTAGCC | AGGTCCTTCGTGTAATAGGTGTC |
| IGF2BP3 | TATATCGGAAACCTCAGCGAGA | GGACCGAGTGCTCAACTTCT |
| IL6 | ACTCACCTCTTCAGAACGAATTG | CCATCTTTGGAAGGTTCAGGTTG |
| β-actin | CATGTACGTTGCTATCCAGGC | CTCCTTAATGTCACGCACGAT |
